# A single baroreceptor unit consists of multiple sensors

**DOI:** 10.1038/s41598-021-02563-x

**Published:** 2021-11-30

**Authors:** Jun Liu, Nana Song, Yufang Wang, Jerome Walker, Jerry Yu

**Affiliations:** 1 Robley Rex VA Medical Center, Louisville, KY 40206 USA; 2 Department of Pulmonary Medicine, University of Louisville, ACB-3, 550 S. Jackson St., Louisville, KY 40292 USA

**Keywords:** Neuroscience, Sensory processing, Physiology, Circulation

## Abstract

Arterial baroreceptors (BRs) play a vital role in the regulation of the cardiopulmonary system. What is known about how these sensors operate at the subcellular level is limited, however. Until recently, one afferent axon was considered to be connected to a single baroreceptor (one-sensor theory). However, in the lung, a single airway mechanosensory unit is now known to house many sensors (multiple-sensor theory). Here we tested the hypothesis that multiple-sensor theory also operates in BR units, using both morphological and electrophysiological approaches in rabbit aortic arch (in whole mount) labeled with Na^+^/K^+^-ATPase, as well as myelin basic protein antibodies, and examined microscopically. Sensory structures presented in compact clusters, similar to bunches of grapes. Sensory terminals, like those in the airways, formed leaf-like or knob-like expansions. That is, a single myelinated axon connected with multiple sensors forming a network. We also recorded single-unit activities from aortic baroreceptors in the depressor nerve in anesthetized rabbits and examined the unit response to a bolus intravenous injection of phenylephrine. Unit activity increased progressively as blood pressure (BP) increased. Five of eleven units abruptly changed their discharge pattern to a lower activity level after BP attained a plateau for a minute or two (when BP was maintained at the high level). These findings clearly show that the high discharge baroreceptor deactivates after over-excitation and unit activity falls to a low discharge sensor. In conclusion, our morphological and physiological data support the hypothesis that multiple-sensory theory can be applied to BR units.

## Introduction

Baroreceptors (BRs), like mechanosensors in the airways^1^, atria, and ventricles^2^, are stretch receptors found in the walls of the blood vessels. Two major arterial BR fields lie in the carotid sinus and aortic arch and play an important role in the regulation of the cardiovascular system by providing beat-by-beat information regarding arterial blood pressure (BP). This information is transmitted to the brain and results in reflex BP and heart rate regulation^3^.

In early morphologic studies of nerve endings in the heart methylene blue and silver impregnation were used to detect sensory receptors. Miller and Kasahara provided an excellent detailed description of various structures in the atria, including complex unencapsulated endings, and end-net structures^4^. Complex unencapsulated endings include compact or diffuse types. Compact ones are from moderate to large myelinated fibers with more spatially circumscribed endings of varying size and form. Diffuse ones have moderate myelinated fibers with many branches and occupy a considerable area. End-net structures, however, are formed by the anastomoses of several branches different myelinated fibers. Similarly, various BR structures are found in animals^5,6^ and humans^7,8^.

In 1935, Nonidez gave a fairly detailed account on BR structures, noting two types of end-formations: diffuse and dense terminals^6^. He described many terminals as delicate rings or small club-shaped dilations. In 1972, Aumonier confirmed the two types of BRs. One spread diffusely over a large area with a net, and the other tightly formed into packed fusiform structures^5^. With confocal microscopy and neural tracers, two types of BRs were later described as “end-net” and “flower spray”^9^. Morphologies of aortic and carotid BRs are strikingly similar, as are BR structures among different species^10^. Anatomically, BRs are connected to two types of afferents: myelinated A fibers (ABRs) and non-myelinated C fibers (CBRs)^11,12^. Comparatively, the former have fast conduction velocity, low threshold, and high regular discharge frequency and sensitivity^12^. Physiologically, afferents from carotid BRs are also classified into type I and type II. Type I has high discharge frequency and sensitivity and a narrow operating range. In contrast, type II has low firing frequency and sensitivity and a wide operating range^13^. Most type I receptors are supplied by large A fibers, whereas type II receptors by small A and C fibers^14^. Type I and II BRs are believed to play an important role in acute regulation of BP and in long term control of mean BP, respectively^15^. Despite extensive studies of the BRs, our knowledge of their mechanosensory transduction mechanism remains incomplete.

Recently, airway mechanosensory units were found morphologically^16^ and physiologically^17^ to consist of multiple-sensors. These sensors interact through encoder switch^18^, including a mechanism of deactivation^19^. Thus, the sensory unit is not merely a transducer, but also a processor that integrates information. Arterial ABRs are similar to slowly adapting receptors (SARs) in the bronchopulmonary system, in their regular discharge characteristics and high sensitivity to distending pressures, and in connection to the myelinated afferent. Here we hypothesized that ABR units also consist of multiple-sensors, and operate with encoder switch and deactivation. Since SAR structures have been successfully studied with anti-Na^+^/K^+^-ATPase (α3 subunit) and myelin basic protein (MBP) antibodies for histochemical staining^16,20–22^, we used the same histochemical techniques to examine the BR units. In addition, we recorded single unit activity from the depressor nerve and examined ABR unit response to over-distending pressure (i.v. phenylephrine). Indeed, our morphological and physiological data support the multiple-sensor theory in BR units. A preliminary account of some of these findings has already appeared^23,24^.

## Methods

Studies were carried out in male New Zealand white rabbits (1.8–2.2 kg). Animals were anesthetized with 20% urethane at 1 g/kg (i.v.), Study protocols complied with Animal Research Reporting of In Vivo Experiments (ARRIVE) guidelines and Guide for the Care and Use of Laboratory Animals published by the United States National Institutes of Health (NIH Publication No. 85-53) and were reviewed by the Institutional Animal Care and Use Committee at University of Louisville and the Robley Rex VA Medical Center.

In morphological studies, pressure sensitive regions were first identified by recording the electrical activity from the depressor nerve and probing the receptive field. Using this method, a definitive sensory region is found by receptor discharge in response to the probe. The depressor nerve responds with a mounting discharge to continuous probing. After identification of the sensitive regions, the rabbit was sacrificed under deep anesthesia by an overdose of saturated KCl (i.v.) to arrest the heart. The aorta was immediately harvested and fixed overnight in a 0.1 M phosphate-buffered solution containing 4% paraformaldehyde (at pH 7.4), similar to the procedures used to identify airway mechanoreceptors^16,20^. The tissues were reacted with a monoclonal antibody (Anti-Na^+^/K^+^-ATPase, α3 subunit; Biomo Res Lab Inc. PA, USA; diluted to 1:200), and then interacted with secondary antibody tagged with fluoroscein (donkey anti-mouse immunoglobulin G; Jackson Immuno Research; diluted at 1:100). After proper treatment, tissue specimens were examined under a fluorescence microscope fitted with a filter for detection of cy3. Some aortic tissues were also incubated with chicken polyclonal anti-myelin basic protein (MBP) (AVES Labs, Inc. OR, USA; diluted to 1:100) for double staining. Sensory receptors were examined at different levels in the tissue by optical sections. These images were saved and the maximum intensities of different optical sections were combined to give a whole view of the receptor structures. Nonspecific staining is ruled out by omitting primary antibody. Negative results were found in the antibody omission studies, confirming the quality of the staining method. Sensory structures were examined by a fluorescent microscope (Olympus system; Model 1X71).

For functional studies, we used the single fiber recording technique in anesthetized rabbits^25^. Briefly, a midline incision was made to expose the trachea, which was then cannulated for mechanical ventilation (Harvard ventilator, model no. 683; South Natick, MA). The chest was opened widely. Positive-end-expiratory pressure (PEEP) was maintained by placing the expiratory outlet under 2–4 cmH_2_O. A common carotid artery and a femoral vein were cannulated for BP measurement and medication, respectively. The depressor nerve (either right or left) was separated into a small bundle, placed on a dissecting platform, and covered with mineral oil. A small slip was isolated from the nerve bundle and placed on recording electrodes, with the main trunk of the depressor nerve intact. The electrodes were connected to a high-impedance probe (Grass HIP5), from which the output signal was amplified (Grass P511). Unit activity signals were displayed on an oscilloscope and recorded along with BP and airway pressure by a Dash IV thermorecorder (Astro-Med, FL, USA). ABRs were identified by their characteristic discharge pattern. Impulse frequency was counted by a rate meter at a bin width of 0.05 s. Unit responses to a bolus intravenous injection of phenylephrine (10 mM, 0.2 ml) were examined. The mean activity was calculated by averaging over 10 s in the control period, and averaging over 5 cardiac cycles at the peak. In some cases, a femoral artery was also cannulated for withdrawal or infusion of blood to assess the unit responses to mechanical changes in BP.

### Statistical analysis

Group data are expressed as mean ± SE. Two-group comparisons were made by paired student t-test using GB-STAT. p < 0.05 was considered to be statistically significant.

### Disclaimer

The contents do not represent the views of the U.S. Department of Veterans Affairs or the United States Government.

## Results

**Figure 1:**
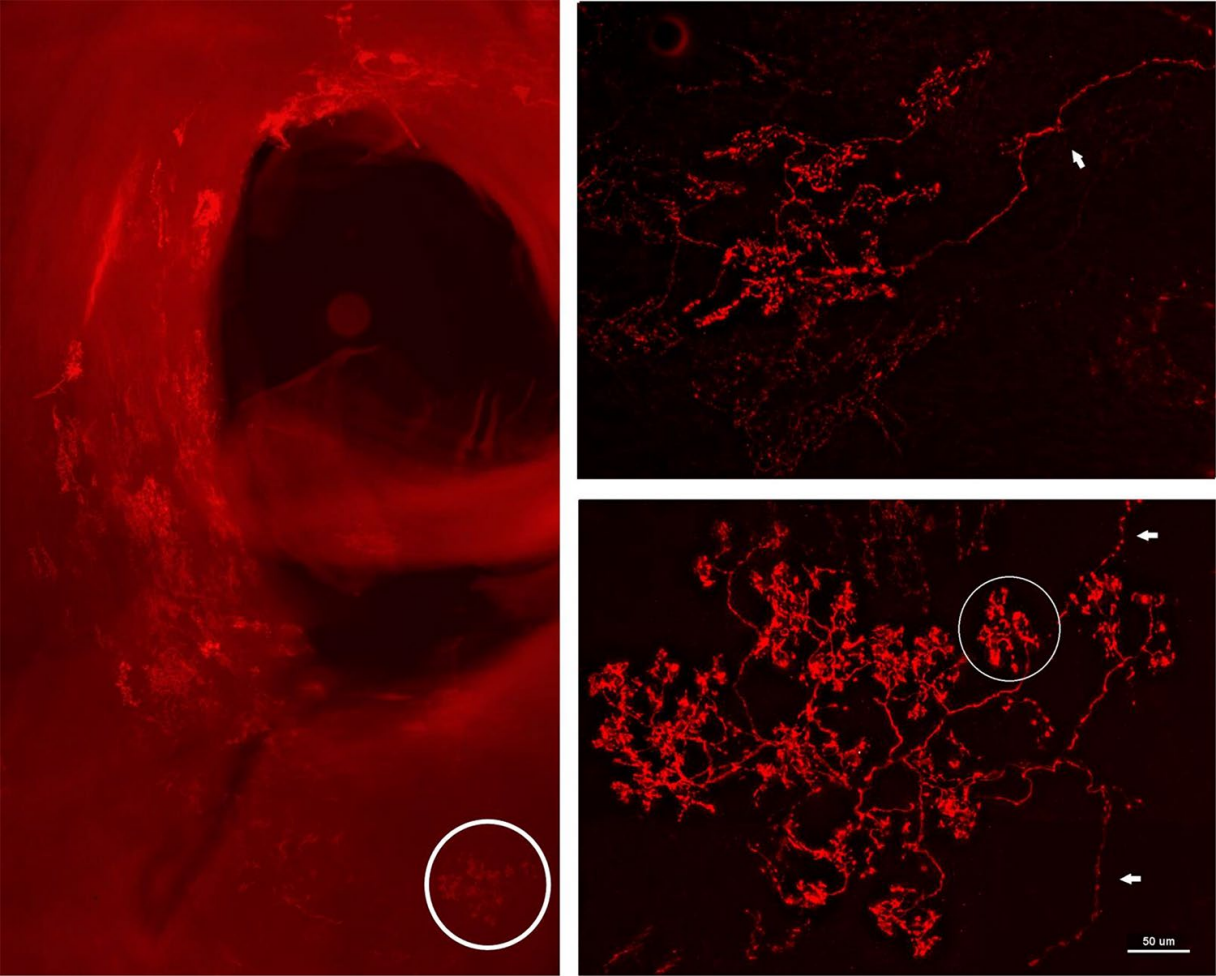
Illustration of baroreceptors in aortic artery. Left: baroreceptor distribution. The sensory receptors are clustered in different areas along the aortic artery. They are more concentrated in the bifurcation origin of the common carotid and subclavian arteries. The white circle shows the BR structure illustrated on the right (bottom). Right: sensory structures presented in compact clusters resembling bunches of grapes. Morphologically, receptor terminals, similar to airway mechanoreceptors, swell to form knob-like or leaf-like end-formations. A single parent axon may connect with multiple sensory receptor structures, indicated by white arrows. The white circle at the right bottom encircles a single BR. The scale bar is 50 µm.

BR structures were found in the aortic arch near the bifurcation or origin of the common carotid and subclavian arteries (Fig. 1, left). The region containing baroreceptors verified by probing the receptive field during electrical recording was used for dissection. Most commonly they were located in the inner adventitia, close to the tunica media (muscular layer), but not in the intimal layer. BR structures varied slightly from one to another, even within the same sensory region. However, their basic structural characteristics were similar, forming a series of divisions that looked plant-like.

**Figure 2:**
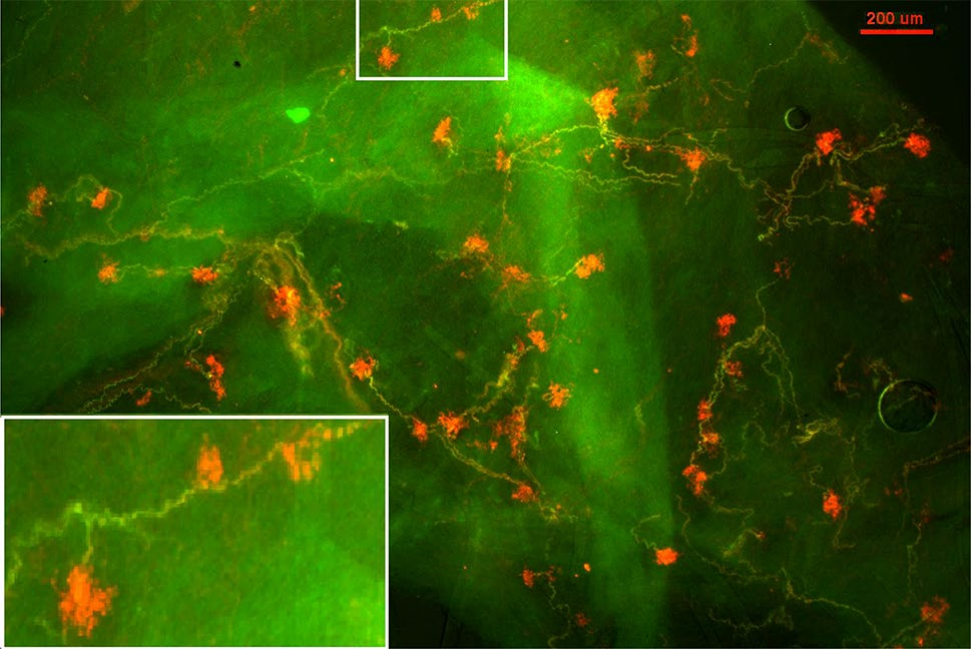
A double staining approach to illustrate BR structures. Na^+^/K^+^-ATPase stains all structures in the sensory unit (red); myelin basic protein (MBP) stains the myelin sheath (green) and co-staining shows yellow. Each red ball shaped end formation (a compact flower spray) is a receptor, demonstrated by a myelinated axon termination. The lower left corner is an enlargement of the structure in the up middle rectangular. The scale bar is 200 µm.

Some BRs were compact (dense) and round or oval in shape, presenting in clusters similar to bunches of grapes (Figs. 1, right, and 2). Figure 2 is a lower power view. Each compact ball-shaped structure is a single baroreceptor that can be identified by the termination of a myelinated axon. In the lower left corner is an enlargement of the structure in rectangular form. It shows at least three receptors connect to a parent axon, indicating multiple sensors, and supporting the multiple-sensor theory.

**Figure 3:**
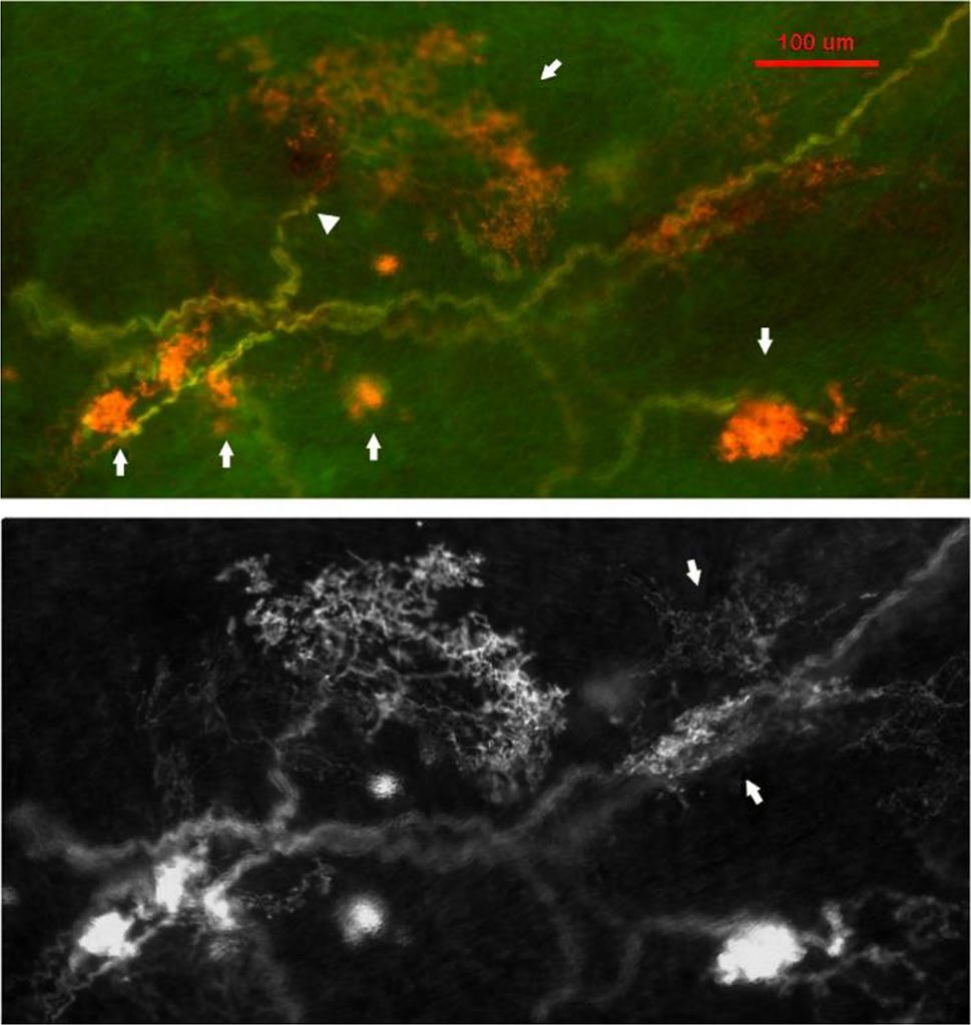
A double staining approach (Na^+^/K^+^-ATPase and MBP) to identify baroreceptor structures. Two types of can be identified: (1) solid, compacted, and ball-like structures (four arrows in upper figure denote each); (2) a much larger, extended diffuse-type structure (denoted by arrows in the upper middle of the figure). Clearly, they differ morphologically. The intensity of the staining is much higher in the former; however, both are connected to myelinated afferents (green axons), which is denoted by an arrowhead for the diffuse-type. The diffuse structure is much more easily observable in the black and white figure (bottom), in which 2 arrows denote 2 other smaller diffuse structures. The scale bar is 100 µm.

**Figure 4:**
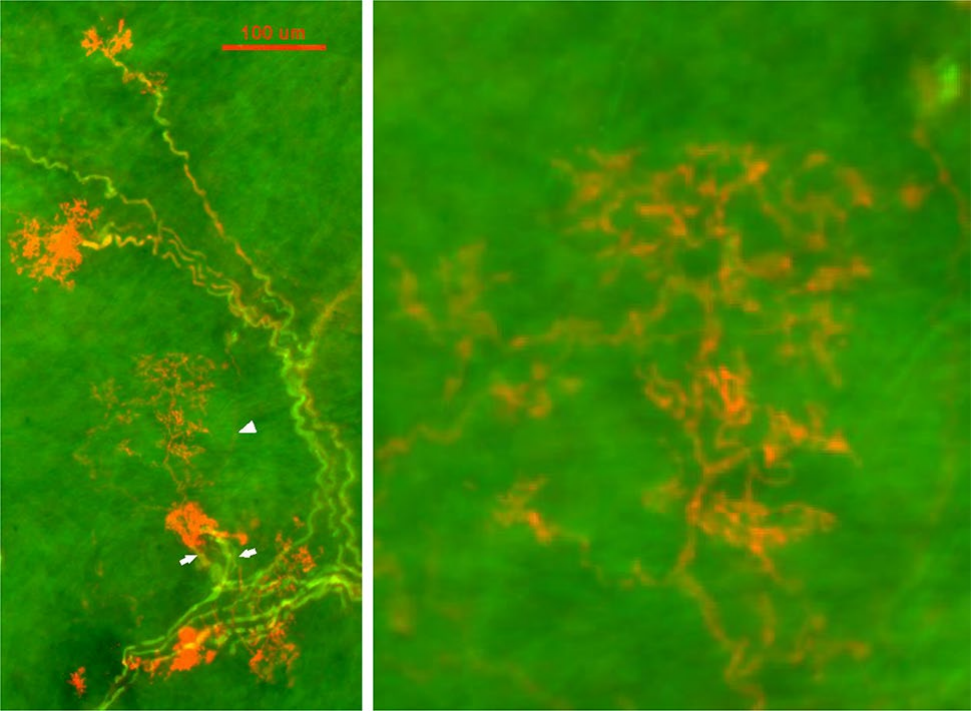
More samples of BR structures with double staining of Na^+^-K^+^-ATPase (red) and myelin basic protein (green), showing different types of morphologies. Some are more concentrated round compact type while others are more extended diffuse type. Again, many sensors may share an axon, forming a unit. On the right is the diffuse type found in the middle of the left picture denoted by an arrow head. Two white arrows indicate myelinated axons for a compact type (left) and a diffuse type (right), respectively. The scale bar is 100 µm.

**Figure 5:**
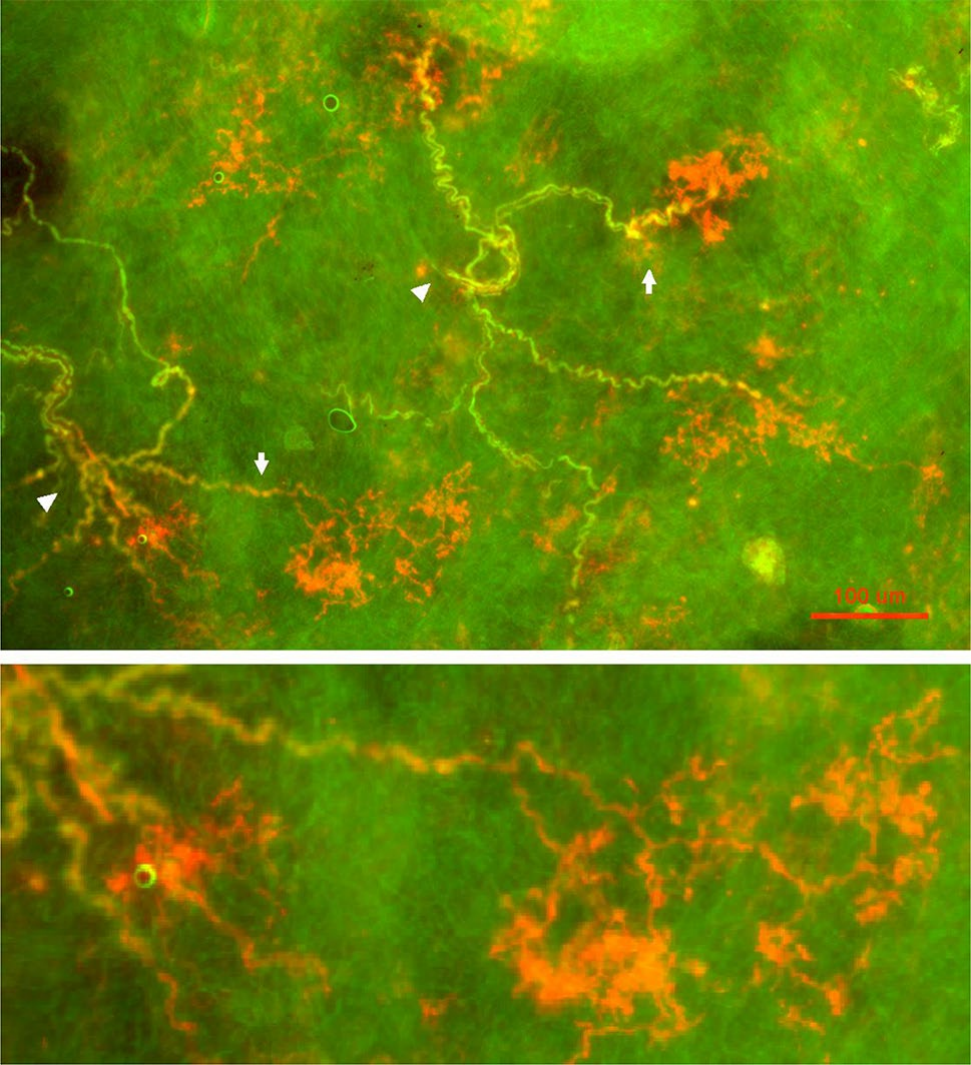
Another example for 2 types of morphology. Two white arrows indicate a diffuse type (left lower) and a compact type (right upper), respectively. Two white arrowheads indicate many axons from different receptors converge. The bottom part is the enlargement of the diffuse type. The scale bar is 100 µm.

In addition to the compact ball shaped structures, other sensory endings were more diffuse, resembling twigs with endings covering a large area (Fig. 3). In Fig. 4, on the right, is the enlarged diffuse structure found in the middle of the left figure. Clearly, it is distinctive from the compact ones. Interestingly, both diffuse and compact sensory endings are connected to myelinated afferents (Figs. 3, 4, 5).

**Figure 6:**
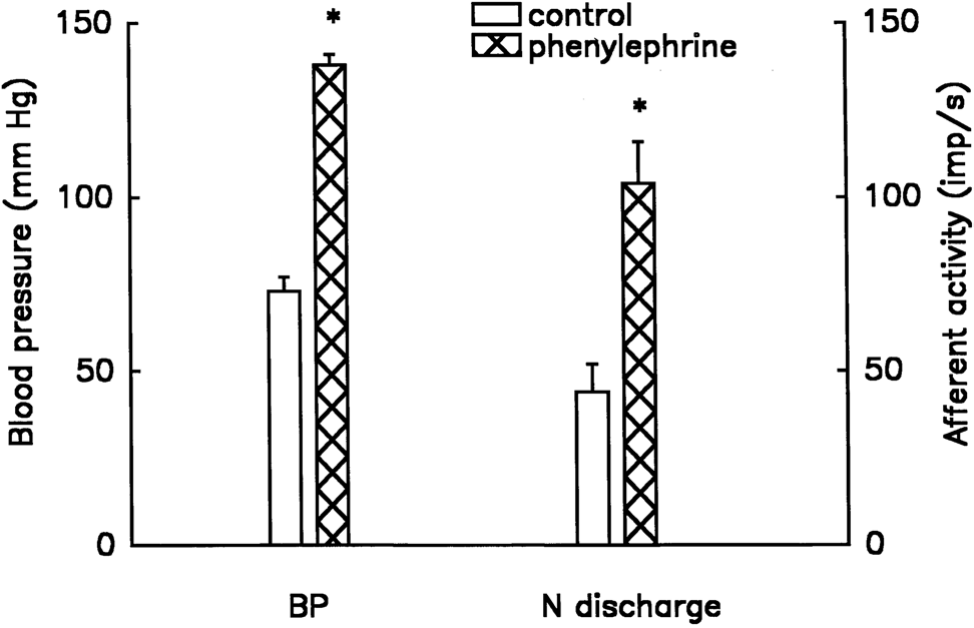
Response of baroreceptor units (n = 11) to intravenous injection of phenylephrine (10 mM, 0.2 ml). Blank and hatched bars are control and after phenylephrine, respectively. *p < 0.001.

In a series of single-unit recordings, bolus injection of phenylephrine (10 mM, 0.2 ml) increased unit activity progressively from 44 ± 8 to 104 ± 12 imp/s (n = 11, p < 0.001) within 13 ± 1 s, as BP increased from 73 ± 4 to 138 ± 3 mmHg (p < 0.0001) (Fig. 6). Five of eleven units evaluated changed their discharge pattern abruptly after the BP attained a plateau for 143 ± 64 s.

**Figure 7:**
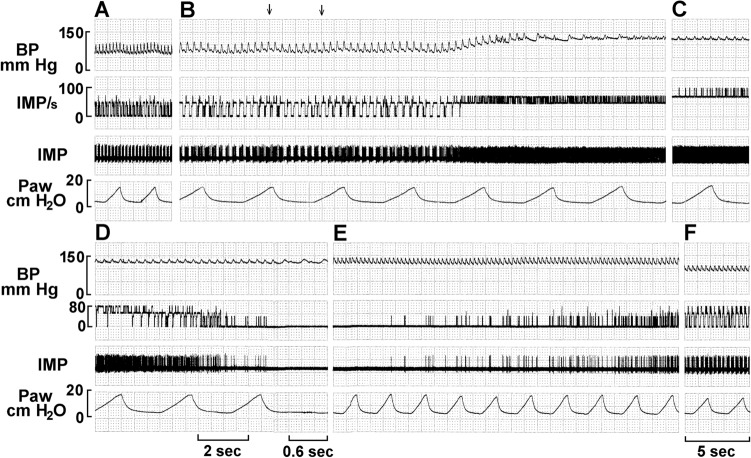
Typical baroreceptor unit response recorded in the left depressor nerve of an anesthetized, open-chest and mechanically ventilated rabbit. Traces are: BP, arterial blood pressure; IMP/s, unit activity, impulses counted at a bin width of 0.05 s; IMP, unit activity; Paw, airway pressure. A, control; B, unit response to intravenous injection of phenylephrine (10 mM, 0.2 ml, indicated by the two arrows on top of the BP trace). Time periods between B–C, C–D, and D–E were 40, 10, and 30 s, and between E–F was 3 min. Please note that the unit discharges at very high frequencies as the BP is maintained at a high level (C); unit activity became irregular before inactivating (D). Please also note that A, E, and F have the same paper speed, so do B, C and D.

Typically, BR activity increased as BP increased in response to phenylephrine injection (Fig. 7). During BP elevation, initial high discharge frequency (end of Fig. 7B,C) then slows, becomes irregular, and subsequently discharges at a low level, or ceases entirely (deactivation) (Fig. 7D). The unit reactivated when BP was gradually lowered (E and F).

**Figure 8:**
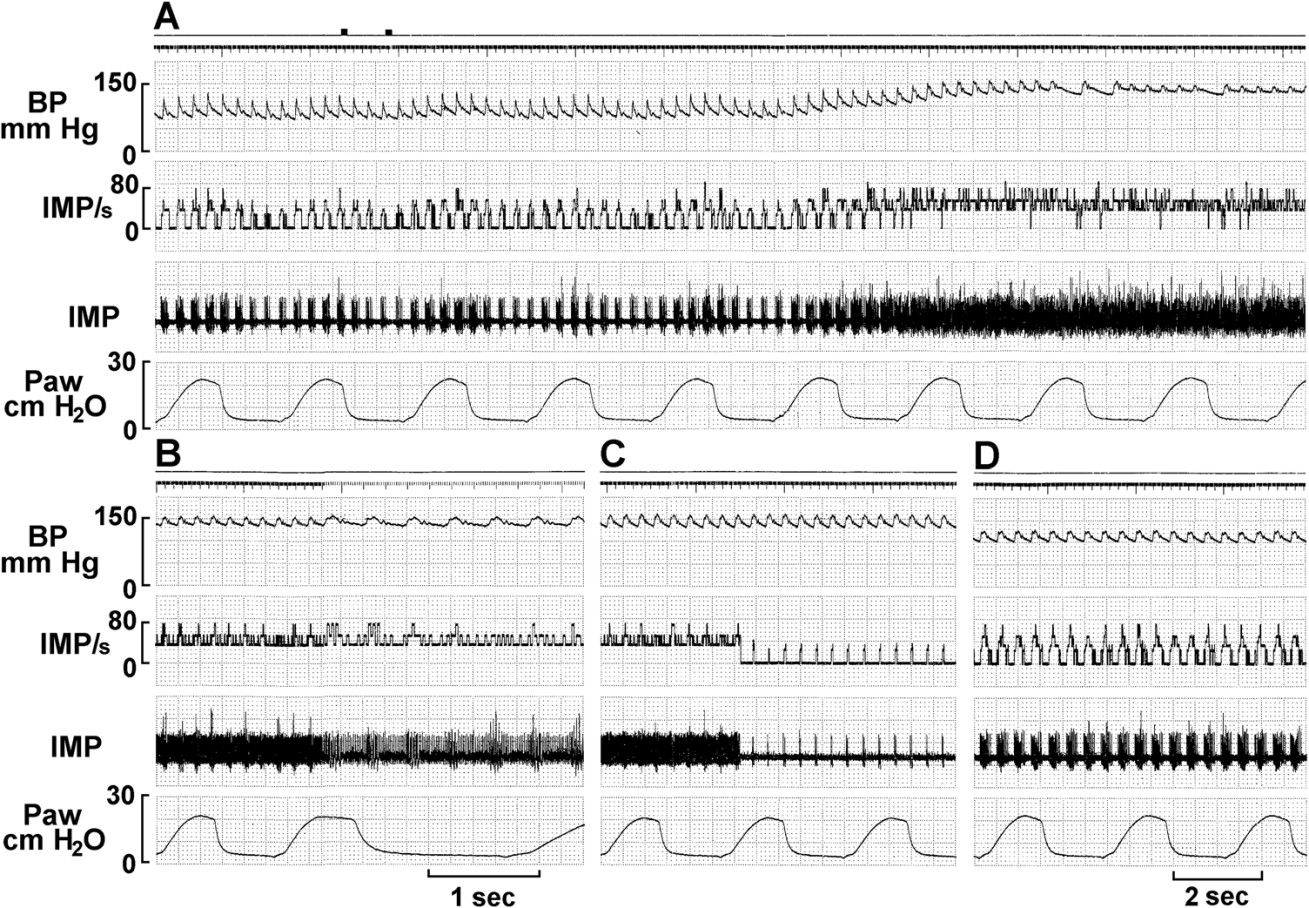
Unit activity of aortic baroreceptors in an anesthetized rabbit. The traces from top to bottom are: arterial blood pressure, impulse activity per second, impulse activity, and airway pressure. The time elapsed between A and B, B and C, and C and D were 50 s, 50 s, and 430 s respectively. The two black markers on the top of figure A indicate injection of a vasopressor (phenylephrine). Please note the baroreceptor activity increases as arterial blood pressure increases following the injection of the vasopressor (A). The unit discharged at higher frequency continuously until 129 s after the injection. Then the activity abruptly decreases (C); it is still discharging in phase with cardiac rhythm. Please note that the discharge frequency is low even though the blood pressure stays high around 150 mmHg. The decreased activity is due to pacemaker switching, indicating that a single baroreceptor unit possesses multiple encoders. The discharge pattern returned to normal 420 s after the pacemaker switching (D).

Evidence a single BR unit can house multiple encoders appears in Fig. 8. The unit discharged at high frequency as BP increased after phenylephrine. In approximately 2 minutes, while BP remains elevated, the discharge frequency abruptly drops (C), indicating an encoder switch, i.e., the unit operation switches from a high discharge encoder to a second low discharge encoder.

**Figure 9:**
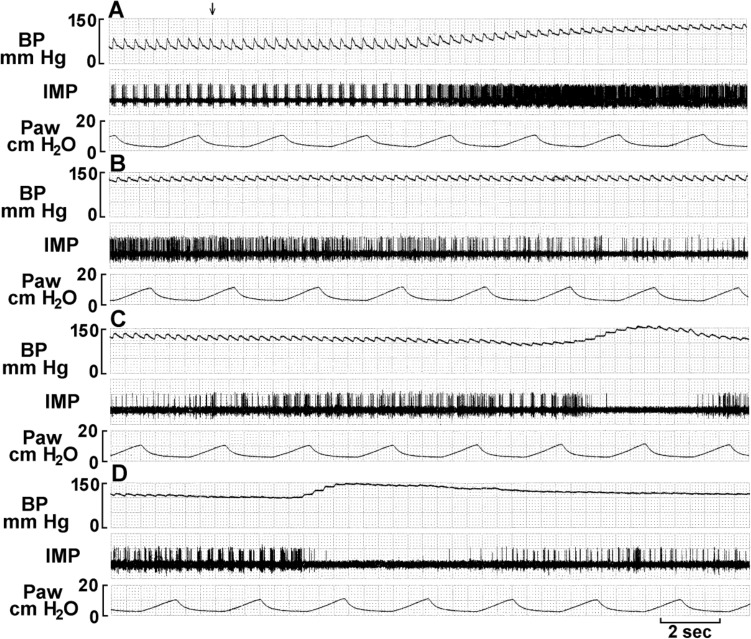
Another baroreceptor unit in response to phenylephrine injection (indicated by the arrow in A). A and B, and C and D are in continuance with a 30 s elapse between B and C. Please note that after inactivated, the unit became active as blood pressure was lowered by withdrawal of blood from the femoral artery (about 20 ml) at the beginning of C. The unit was inactivated again by increasing the blood pressure by injection of the blood into the artery. Such activation and inactivation can be repeated.

**Figure 10:**
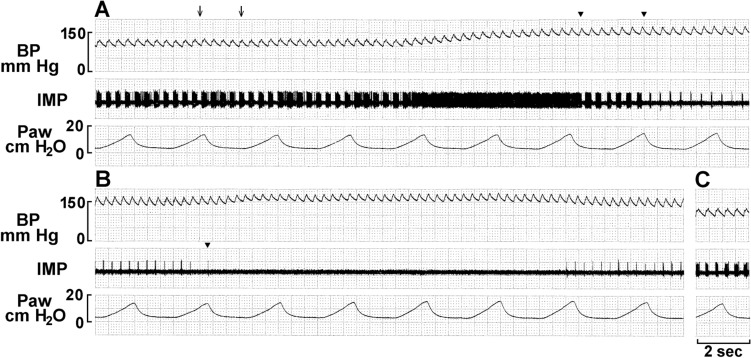
Baroreceptor unit response to phenylephrine injection (2 arrows at the beginning of A). Blood pressure increased approximately 6 s thereafter, during which time the unit deactivated in three steps (denoted by 3 arrow heads at the end of A and beginning of B), indicating at least 3 encoders in this unit. Complete deactivation occurred at the last arrow head, induced by injection of blood into the artery (starting immediately before the last arrow head) and re-activated as blood withdrawn. C is post-control.

After deactivation, sensory units could be reactivated by lowering BP by withdrawal of blood and deactivated again by returning BP to the high level with injection of blood into the artery (Figs. 9 and 10). These data clearly show over-excitation deactivates the BRs.

Figure 10. shows a BR unit with multiple deactivation (3 times) and generates 3 different levels of activities during a sustained increase in BP, demonstrating activity arising from three different sensors. Also notable is any number of sensors may operate within a unit, more than can be readily counted. In Fig. 9, for example, as blood pressure increases unit discharge increases. At the end of recording A, there is a subtle but detectable decrease in activity, which progresses into recording B. Here, unit activity does not show clear deactivation to a discrete lower level, but may represent a series of deactivations, a process involving many sensors. This fits the morphological results that many sensors connect to an afferent, not just 2 or 3 as demonstrated in Figs. 8 and 10.

## Discussion

Our studies provide the first evidence a single arterial ABR unit resembles its counterpart airway SAR unit in possessing multiple sensors. Morphologically, multiple sensors may connect to a single axon, allowing the unit to demonstrate multiple sensor behavior physiologically.

In histochemical studies, BR structures in the aorta, like SARs in the lung^16^, were clearly visualized after reacted with antibodies against Na^+^/K^+^-ATPase and MBP. This supports BRs and SARs having the same chemical composition. Previous confocal microscopy work showed two types of BR structures: flower spray and end-net^9^. Our present studies further demonstrated two types (compact and diffuse) of flower spray morphology (Figs. 3, 4, 5), corresponding with compact and diffuse complex unencapsulated endings reported in the atria^4^. We believe our diffuse sensors are not the end-net BR structures because they are more plant-like, the end-net seldom contains leaf-like and knob-like end formations. In a recent report, four sensory structure types have been described: large flower spray, small flower spray, large end-net, and small end-net^26^. We do not know the exact correspondence of our structures to these. Our compact and diffuse structures could be small and large flower spray endings, respectively. However, compact and diffuse types have a clear different morphology and are likely different. A clear differentiation between morphologies obtained by different techniques [DiI^9^ versus histochemical staining (present study)] is difficult. And apart from these terminological considerations, there remains no direct correlation between these morphological studies, such as the “compact” and “diffuse” types, and the physiological single unit recordings. Only by integrating and reconciling their morphology and function can the sensory mechanisms in baroreceptors be fully understood.

A particular difficulty when trying to identify the specific structure and function in the BRs is that they are clustered within the same region. This disallows selectively blocking one without affecting the other anatomically for physiological studies. Isolating the two structural types for a physiological study to clarify this issue likely awaits new genetic or pharmacologic methods. In the meantime, here we show an association between BR morphology and physiology in that multiple sensors are connected with an afferent axon. And two types of myelinated sensors (compact and diffuse; Figs. 3, 4, 5) potentially share an afferent axon. This new information should facilitate further physiological studies and re-interpretation of BR unit behaviors. For example, electrical activities of ABR units demonstrate rapidly and slowly adapting components in response to a constant pressure stimulation^27^, as do mechanosensory units in the airway^1^. The compact and diffuse sensors may be rapidly and slowly adapting receptors, respectively. Nevertheless, we believe, as Cheng’s group^26^, that flower spray sensors likely sense beat-by-beat changes in BP, because physiologically recorded SARs in the airway are flower spray in morphology, either by DiI tracer^28^ or by histochemical staining^21^ techniques. However, in a recent study, contradictory results were obtained. The authors found that ablation of PIEZO2 neurons eliminated “the end-net structures” and the baroreceptor reflex, while ablation of MC4R neurons eliminated the flower spray structures, but had no effect on baroreflex function^29^. However, there is no recording of BR activity in that study.

More importantly, our histochemical data show a single myelinated axon may connect with multiple receptors clustered in the sensory region of the aortic arch. Figures 1, 2, 3, 4 and 5 show a single sensory axon connected with many individual receptor structures. The receptors interconnect to form a cluster. If a receptor is an encoder, which is the basic device that generates action potentials (as in the SAR), such a morphological arrangement suggests that a single BR unit consists of multiple encoders^1^. This is an important new concept, which changes our view on BR functions. Indeed, our morphological data are supported by physiological studies.

Physiologically, at least three types of baroreceptors have been described as large myelinated, small myelinated, and non-myelinated receptors^15^. While linking morphology with physiology requires further investigation, our physiological studies support the morphological data and support multiple-sensor theory. In single-unit recordings, BR activity increased progressively as BP increased, and 5 out of 11 ABR units (45%) decreased abruptly (deactivation) after the BP attained a plateau (Figs. 6, 7, 8, 9 and 10). BR activity became slow first (indicating multiple encoders) and subsequently ceased. The unit could be re-activated by lowering BP and deactivated again by raising BP. These BR behaviors are the same as SARs in response to over-excitation.

It is important to note that multiple sensors are verified in the muscle spindle^30^ and Golgi tendon organs^31^ without involving encoder deactivation. These sensors may be activated singly or in combination, and interactions between them can be studied without needing deactivation to prove multiple sensors. In cardiopulmonary sensory units, however, changes in pressure activate all sensors in the unit. To prove multiple sensors in a unit requires assessing unit activity before and after removing an active sensor.

In lung units, overly-excited slowly adapting receptors (SARs) may deactivate, either in response to chemical stimulation (ouabain^32^) or mechanical stimulation (hyperinflation of the lung with constant pressure^19^, or cyclic pressure^33^). After deactivation, when the stretch of the unit is unloaded, the SAR may reactivate. Evidence that a high frequency sensor may shut off, with unit activity falling to a low frequency sensor due to pacemaker switch (or encoder switch), comes from findings where high frequency activity can be selectively blocked by injection of lidocaine into the sensory receptive field (see Fig. 2 in Ref.^19^). In SAR units, sensors may be separated from several millimeters up to centimeters, allowing selective blockade^18^. Such experiments cannot be repeated in BR units because they cluster together (Figs. 1, 2). However, over-excitation-related deactivation and reactivation of the baroreceptors are best illustrated in Figs. 9 and 10. Taken together, ABRs and SARs have similar morphologies and afferent properties. Both are stretch receptors. Over-excitation leads to deactivation in both. While the evidence that multiple level activities arise from different sensors operating within a single BR unit is not direct, it can reasonably be inferred. At the same BP, 3 different discrete levels of activities (Fig. 10) cannot be explained by the one-sensor theory, but can by the multiple-sensor theory.

On careful review of the literature, the deactivation phenomenon in BRs is found in many reports. Some authors noticed a complete shut-off of baroreceptor activity on exposure to sustained pressures. For example, Biscoe et al. (Fig. 4 of Ref.^34^) described baroreceptor deactivation with saline injection into the carotid sinus of sheep fetus. There was initial increased activity followed by total deactivation of some BR units after intra-sinus pressure was increased acutely with 3 ml saline injection. After a period of inactivity, the unit resumed firing at the basal rate. This phenomenon could be reproduced by withdrawing and reinjecting blood from and to the animal. Hence the authors concluded that this switch-off of activity was not secondary to local PaO2 changes caused by saline injection. But no satisfactory explanation for this phenomenon was provided. Similarly, in an aortic ABR unit study (Fig. 8 of Ref.^35^), Angell James stated: “At a certain pressure the relationship between the impulse frequency and intra-aortic pressure ceased to be linear and this pressure is described as the point of inflexion… The impulse frequency at the point of inflexion was not necessarily the maximum frequency attained in any given fibre; in some fibres, at least, the impulse frequency continued to increase with pressure whereas in others the impulse frequency either remained constant, diminished, became intermittent or decreased to zero.”^35^. This is a clear description of deactivation. After deactivation, some units operate at lower activity levels while others shut off completely. In other studies, there is a clear deactivation of aortic CBR unit in rabbits (see Fig. 5 of Ref.^11^). The unit activity at mean arterial pressure of 130 and 150 mmHg is lower than at 120 and 140 mmHg, respectively. Similar observations are found in cat carotid BRs when comparing the activities at 160 mmHg with 260 and 300 mmHg (see Fig. 7 of Ref.^36^). Indeed, the occurrence of deactivation is high, which is demonstrated by 3 of the 4 units illustrated in a study of dog aortic BRs (Fig. 4 of Ref.^37^). Since multiple sensors lying within a single mechanosensory unit are found in sheep^34^, cat^36^ and dog^37^ ABR units (see above), and also in bronchopulmonary units in various animal species (Ref.^38^), including rats and mice. We believe that multiple-sensors in a single BR unit is a common mechanism not specific to rabbits.

The BR unit is not merely a transducer, but also a processor that integrates information. Unit activity recorded in the depressor nerve results from the integration of the outputs from its active encoders. Each encoder generates action potentials, sending signals through daughter afferent axons for interaction (or integration). After integration, the unit generates a unified train of action potentials to the CNS through their parent axon for further signal processing. Significant information integration occurs at the intra-encoder and inter-encoder levels. Confirmation of the multiple-sensor theory helps our understanding of the sensory signal processing mechanisms at the cellular level. Such multiple-sensor mechanisms operate in the visceral organs, the lung^1^, cardiovascular (present studies), and gastrointestinal systems^39^.

Lastly, a sustained increase in BP increases baroreceptor activity initially, which declines over time. This is termed adaptation, first described by Bronk and Stella in 1934, and subsequently expanded upon by Landgren in 1952^27^. Adaptation and re-setting are two recognized characteristics of BRs. Both are related to relaxation of viscoelastic coupling elements, leading to a reduction of strain at the receptive fields^3^. Re-setting is defined as a shift of the stimulus–response curve. The exact mechanism of re-setting has not been elucidated, although many mechanisms, including mechanical and ionic factors, are believed to be responsible^40^. We are now able to offer another mechanism, sensory deactivation as part of the re-setting.

In summary, our approach provides an excellent tool to explore the structure of BR units and demonstrates the existence of two types of ABR morphologies (flower spray and network). We believe these ABR units sense beat-by-beat information for cardiovascular regulation. Furthermore, we demonstrated that a single afferent axon may be connected with many BRs to form a sensory unit, supporting the multiple-sensor theory. Thus, this arterial ABR unit is not merely a transducer, but also a processor that integrates information before sending it to the brain for further interpretation.
